# Tra2beta-Dependent Regulation of RIO Kinase 3 Splicing During Rift Valley Fever Virus Infection Underscores the Links Between Alternative Splicing and Innate Antiviral Immunity

**DOI:** 10.3389/fcimb.2021.799024

**Published:** 2022-01-19

**Authors:** Luke Adam White, Thomas C. Bisom, Hunter L. Grimes, Miyuki Hayashi, Jean-Marc Lanchy, J. Stephen Lodmell

**Affiliations:** ^1^ Division of Biological Sciences, University of Montana, Missoula, MT, United States; ^2^ Department of Chemistry and Biochemistry, University of Montana, Missoula, MT, United States; ^3^ Center for Biomolecular Structure and Dynamics, University of Montana, Missoula, MT, United States

**Keywords:** Rift Valley fever virus, alternative splicing, innate immunity, RNA viruses, viral infection, splicing factors

## Abstract

Rift Valley fever virus (RVFV) is an emerging pathogen that has potential to cause severe disease in humans and domestic livestock. Propagation of RVFV strain MP-12 is negatively impacted by the actions of RIOK3, a protein involved in the cellular immune response to viral infection. During RVFV infection, RIOK3 mRNA is alternatively spliced to produce an isoform that correlates with the inhibition of interferon β signaling. Here, we identify splicing factor TRA2-β (also known as TRA2beta and hTRA2-β) as a key regulator governing the relative abundance of RIOK3 splicing isoforms. Using RT-PCR and minigenes, we determined that TRA2-β interaction with RIOK3 pre-mRNA was necessary for constitutive splicing of RIOK3 mRNA, and conversely, lack of TRA2-β engagement led to increased alternative splicing. Expression of TRA2-β was found to be necessary for RIOK3’s antiviral effect against RVFV. Intriguingly, TRA2-β mRNA is also alternatively spliced during RVFV infection, leading to a decrease in cellular TRA2-β protein levels. These results suggest that splicing modulation serves as an immune evasion strategy by RVFV and/or is a cellular mechanism to prevent excessive immune response. Furthermore, the results suggest that TRA2-β can act as a key regulator of additional steps of the innate immune response to viral infection.

## 1 Introduction

Rift Valley fever virus (RVFV) is a mosquito-borne bunyavirus (Order *Bunyavirales;* Family *Phenuiviridae*) (Adams et al., 2017) that is endemic to Africa and the Arabian Peninsula and causes disease in livestock and humans. In livestock, RVFV causes abortive pregnancies and severe illness in young animals, while in humans it can cause a variety of symptoms, from mild flu-like symptoms to liver damage, blindness, hemorrhagic fever, and death (Daubney et al., 1931; Bird et al., 2009; Ikegami and Makino, 2011; Grossi-Soyster and LaBeaud, 2020). There is also evidence that RVFV increases the probability of miscarriage in infected women (Baudin et al., 2016). Its mosquito vectors, species in the *Aedes* and *Culex* genera, are predicted to expand their range in response to climate change (Samy et al., 2016; Ryan et al., 2019; Iwamura et al., 2020), making RVFV a potential threat beyond Africa and the Arabian Peninsula. Because of its potential for severe illness and spread, RVFV is listed as a Category A overlap select agent by the CDC/USDA. There is currently no licensed human vaccine or proven drug treatment for RVFV, and a deeper understanding of its replication and interaction with the host will be important for developing strategies to combat RVFV disease.

The innate immune response against RNA virus infections, including RVFV, is activated through RNA-detecting pattern-recognition receptors in mammalian cells such as RIG-I and MDA5 and culminates in the activation of type-I interferon (IFN) and other cytokine production (Kato et al., 2006; Kuri et al., 2010; Ermler et al., 2013; Weber et al., 2013). In reports from other laboratories as well as recent work in our group, the relatively understudied Rio kinase 3 (RIOK3) has been described as a key, yet enigmatic, member of the antiviral response pathway(s). In our system (Havranek et al., 2021) and in the reports of Feng, et al. (Feng et al., 2014) and Willemsen, et al. (Willemsen et al., 2017), RIOK3 was shown to be involved in activation of the innate immune response somewhere downstream of RIG-I. Curiously, in other systems RIOK3 has also been implicated in deactivation of MDA5 and RIG-I (Takashima et al., 2015; Shen et al., 2021), ascribing RIOK3 a role in muting the innate immune response. Additionally, different viruses have different responses to RIOK3 depletion, with Rift Valley fever virus, hepatitis C virus, and influenza A virus replicating faster in the absence of RIOK3 (Willemsen et al., 2017; Gokhale et al., 2020; Havranek et al., 2021), and Zika virus, Dengue virus, and measles virus replicating slower (Takashima et al., 2015; Gokhale and Horner, 2017). Thus, RIOK3 may act in dual roles, both as an effector and an inhibitor of innate immune responses to viral infection, depending on the cell type, virus type, and the immune pathway that has been activated.

Transcriptomics studies have identified widespread changes in the gene expression and alternative splicing of host genes during RNA virus infection (Boudreault et al., 2016; De Maio et al., 2016; Hu et al., 2017; Gokhale et al., 2021). Recently we observed that many mRNAs were alternatively spliced upon infection with RVFV, among them RIOK3 (Havranek et al., 2019). Alternative splicing, which has been observed for virtually every gene in vertebrates, allows for expression of a diverse range of proteins from a given gene (Leff et al., 1986; Black, 2003; Manning and Cooper, 2017). Because potential splice sites are abundant in mRNA, splice site selection is largely controlled by the carefully regulated expression of splicing factors, which bind to specific regions of pre-mRNA and enhance or suppress potential splice donor or acceptor sites en route to production of the mature mRNA (Long and Caceres, 2008; Jangi and Sharp, 2014; Leclair et al., 2020). Identifying splicing factors and the RNA motifs to which they bind can be an essential step in understanding how gene expression is controlled in different conditions, including in response to viral infection.

**Figure 1:**
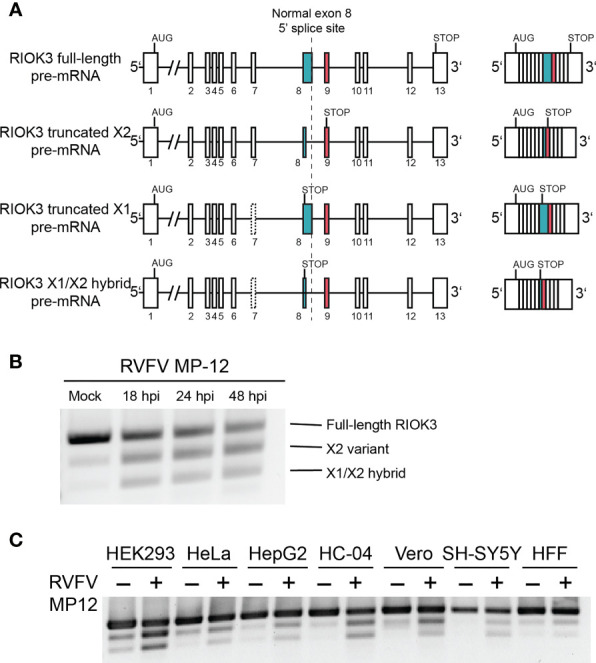
RIOK3 mRNA is alternatively spliced during RVFV infection in multiple cell types. **(A)** Schematic illustration of RIOK3 splice sites observed. Single lines are introns, while boxes are exons. **(B)** RT-PCR visualized by agarose gel showing alternative splicing of RIOK3 pre-mRNA after infection with RVFV MP-12 at MOI = 1. **(C)** RT-PCR showing RIOK3 alternative splicing in multiple cell lines in response to RVFV MP-12 infection. HEK293 (human embryonic kidney), Hela (human cervical carcinoma), HEPG2 (human hepatoma), HC-04 (human hepatoma), Vero (African green monkey kidney), SH-SY5Y (human neuroblastoma), and HFF (human foreskin fibroblast) were used.

RIOK3 mRNA is expressed as four major splice isoforms evidenced by RNAseq (Havranek et al., 2019), which we call full-length (FL), X2, X1, and X1/X2 hybrid (**Figure 1A**). FL contains the entire open reading frame including 13 complete exons for the full-length protein, while X2 utilizes an alternative splice site in exon 8, resulting in a premature termination codon (PTC) in exon 9. X1 excludes exon 7, resulting in a PTC in exon 8. The X1/X2 hybrid has the X2 alternative splice site and a skipped exon 7. The most abundant RIOK3 splice isoform in unstimulated HEK293 cells in culture is FL, while in infected cells X2 predominates. If translated, X2 would lack a large portion of its putative kinase domain and would thus likely be inactive as a kinase. Recently, we also found that constitutive RIOK3 splicing is required for the activation of innate immunity, indicating that the splicing of RIOK3 may be an essential step for regulation of its activity (Havranek et al., 2021).

In this work, we demonstrate that constitutive versus alternative RIOK3 mRNA splicing is controlled by the splicing factor TRA2-β, and that RIOK3 mRNA is alternatively spliced to an alternative isoform in response to RVFV infection in multiple cell types. We also show that RIOK3 alternative splicing occurs shortly after the innate immune response is mobilized to produce interferon. Additionally, we describe that TRA2-β itself is alternatively spliced during RIG-I activation, suggesting that differential expression and processing of this splicing factor may be crucially important to innate immune activation and/or modulation.

## 2 Materials And Methods

### 2.1 Viruses, Cell Culture, and Infections

The MP-12 vaccine strain of RVFV was kindly provided by Brian Gowen (Utah State University, Logan, UT, USA). Manipulations of the viruses used in this study are compliant with both the Institutional Biosafety Committee at the University of Montana, Missoula, and NIH requirements in regard to their handling under BSL2 containment conditions.

All cells were incubated at 37°C and 5% CO_2_. HEK293 (BEI Resources), HeLa (ATCC), HFF-1 (provided by Brent Ryckman, University of Montana, Missoula, MT, USA), and Vero (ATCC) cells were cultured in Dulbecco’s Modified Eagle Medium (DMEM) supplemented with 10% fetal bovine serum (FBS) and penicillin/streptomycin (P/S). HepG2 (provided by Brooke Martin, University of Montana, Missoula, MT, USA), HC-04 (BEI Resources), and SH-SY5Y (provided by Stefan Stamm, University of Kentucky, Lexington, KY, USA) cells were cultured in a 1:1 mixture of DMEM and F12 media, supplemented with 10% FBS and P/S. For experiments using RVFV MP-12, cells were grown to 80-90% confluency, washed with PBS, and overlaid with virus at multiplicity of infection (MOI) of 1. Cells were incubated with virus for one hour, then media was replaced with growth media supplemented with 2% FBS and P/S.

TCID_50_ experiments were carried out as previously described (Smith et al., 2019).

### 2.2 Plasmids and Cloning

The TRA2-β overexpression vector and pSpliceExpress minigene were gifts from Stefan Stamm (University of Kentucky, Lexington, KY, USA). The RIOK3 minigene was constructed by amplifying the RIOK3 ORF from whole genomes extracted from HEK293 cells and inserting it into pSpliceExpress. Mutant minigenes were generated by overlap extension PCR. All clones were verified by sequencing.

### 2.3 Reverse Transcription, PCR, and qPCR

Total RNA was extracted using TRIzol (Thermo Fisher Scientific) and RNA was reverse transcribed using Maxima H Minus Reverse Transcriptase (Thermo Fisher Scientific) with random hexamers according to the manufacturer’s instructions. Standard PCR was carried out using Phusion Flash Hi-Fidelity PCR Master Mix (Thermo Fisher Scientific). Products were run on a 1% agarose gel and visualized using a Molecular Imager Gel Doc XR+ instrument (Bio-Rad). qPCR was performed using the CFX Connect Real-Time PCR Detection System (Bio-Rad). RNA levels were normalized to GAPDH. Relative fold change in expression was calculated using the ΔΔCT method (Livak and Schmittgen, 2001).

See **Table 1** for primers and oligos used in this study.

**Table 1:** List of all primers and morpholino oligonucleotides used in this study.

Name	Sequence (5’ to 3’)
F_RIOK3_Endogenous	CCGGTTCCCACTCCTAAAAAGGGC
R_RIOK3_Endogenous	CCAGCATGCCACAGCATGTTATACTCAC
F_TRA2B	AGGAAGGTGCAAGAGGTTGG
R_TRA2B	TCCGTGAGCACTTCCACTTC
F_RIOK3_Minigene	GACCCACAAGCATGGAGGATGA
R_RIOK3_Minigene	CCAGTTGTGCCAATGAAGAGTTTGA
F_IFNB	AAACTCATGAGCAGTCTGCA
R_IFNB	AGGAGATCTTCAGTTTCGGAGG
F_GAPDH	GTCTCCTCTGACTTCAACAGCG
R_GAPDH	ACCACCCTGTTGCTGTAGCCAA
RIOK3_FL_qPCR_F	GTCTGTTGTCTTTCATGCATATGGAGG
RIOK3_FL_qPCR_R	TGCCCACATGCGGATCTT
RIOK3_Exon1_F	GCCTTCATTCCCGAATGGATCTGGTAG
RIOK3_Exon2_R	GCCAGCTGTTCACTCATTACATCAGCC
RIOK3_X2_qPCR_F	TGCCATCAAGAATGCAGAGA
RIOK3_X2_qPCR_R	TAACTGCCGCATCAAATGAA
TRA2B_Morpholino	ACTTCTTTACCCTGTATATATTTTCCTCTA

### 2.4 Western Blotting

Cells were collected and lysed in radioimmunoprecipitation assay (RIPA) buffer with protease inhibitors (10mM Tris-HCl pH 8.0, 140mM NaCl, 1mM EDTA, 0.5mM EGTA, 1% Triton X-100, 0.1% sodium deoxycholate, 0.1% SDS). Lysates were clarified and subsequently separated by SDS-PAGE on 10% polyacrylamide and wet transferred to PVDF. The membrane was blocked with 5% milk solution in Tris-buffered saline Tween 20 (TBST) at room temperature, and primary antibody was added at a dilution of 1:1000 in milk buffer. Secondary antibody was added at a dilution of 1:10,000 in milk buffer. Following each antibody incubation, the membrane was triple rinsed with TBST. Chemiluminescent visualization of blots was carried out using visualization solution made up of two buffers (buffer 1: 2.5 mM luminol, 0.396 mM coumaric acid, and 100 mM Tris-HCl pH 8.5; buffer 2: 0.0192% hydrogen peroxide, 100 mM Tris-HCl pH 8.5) mixed immediately before visualization.

The following primary antibodies were used: GAPDH loading control antibody MA5-15738 (Thermo Fisher Scientific), anti-RIOK3 SAB1406721 (Sigma), and anti-TRA2-β antibody ab31353 (Abcam). HRP-conjugated secondary antibodies used were anti-Mouse IgG peroxidase antibody produced in goat A2554 (Sigma) and anti-Rabbit IgG peroxidase antibody produced in goat A0545 (Sigma).

### 2.5 Transfection

Plasmid transfections were performed on HEK293 cells using Lipofectamine 2000 as per the manufacturer’s instructions (Thermo Fisher Scientific, Waltham, USA). Morpholino oligos were synthesized by Gene Tools and transfected using Endo-Porter according to the manufacturer’s instructions (Gene Tools).

## 3 Results

### 3.1 RIOK3 mRNA Is Alternatively Spliced During Infection and in Multiple Cell Types

In cell culture, RIOK3 mRNA is expressed as four major splice isoforms termed full-length (FL), X2, X1, and X1/X2 hybrid whose abundances depend on cell treatment and/or cell type (**Figure 1A**). FL represents the constitutively spliced mRNA that is abundant in non-immune-activated cells and contains the entire coding sequence for the full length RIOK3 protein. The X2 isoform is produced due to an alternative 5’ splice site selection event in exon 8, which leads to a PTC in exon 9. X1, the least abundant isoform that is not readily identified *via* agarose gel, contains a skipped exon 7. Additionally, we observe a hybrid X1/X2 splicing event where both exon 7 is omitted and the alternative splice site in exon 8 is observed.

RIOK3 was alternatively spliced in HEK293 cells during the course of RVFV strain MP-12 infection. As early as 18 hours post infection, abundance shifts from primarily FL to X2, with some increase in X1/X2 hybrid (**Figure 1B**). RIOK3 protein level, visualized *via* western analysis (**Supplementary Figure 1**), was slightly increased immediately following RVFV infection, followed by a slow decrease out to 48 hours post infection (hpi). The lack of dramatic changes in full length RIOK3 protein level was not unexpected because we previously demonstrated that alternative splicing of RIOK3 mRNA itself correlated with rapid mitigation of innate immune responses (Havranek et al., 2021). The RIOK3 constitutive-to-alternative splicing pattern was also consistently observed during RVFV infection of multiple cell lines, including HEK293 (human embryonic kidney), HeLa (human cervical carcinoma), SH-SY5Y (human neuroblastoma), HepG2 and HC-04 (human hepatoma), HFF-1 (transformed human foreskin fibroblasts), and Vero (African green monkey kidney) (**Figure 1C**).

### 3.2 Alternative Splicing of RIOK3 mRNA Occurs After IFNB Activation

We previously showed that altering the balance of FL to alternatively spliced RIOK3 mRNA causes ineffective activation of innate immune responses (Havranek et al., 2021). Because overstimulation of innate immunity can lead to cell death and tissue damage (reviewed in Rock and Kono, 2008) and can lead to autoimmune disease (Rodero and Crow, 2016), we hypothesized that RIOK3 splicing may modulate the immune/inflammatory response after an initial robust reaction to pathogen incursion. In particular, if proteins synthesized from the different mRNA isoforms performed distinct functions, the relative abundances of FL, X2 and X1 could provide a mechanism for rapid tuning of the innate immune response and could prevent unwarranted collateral cell damage and apoptosis.

**Figure 2:**
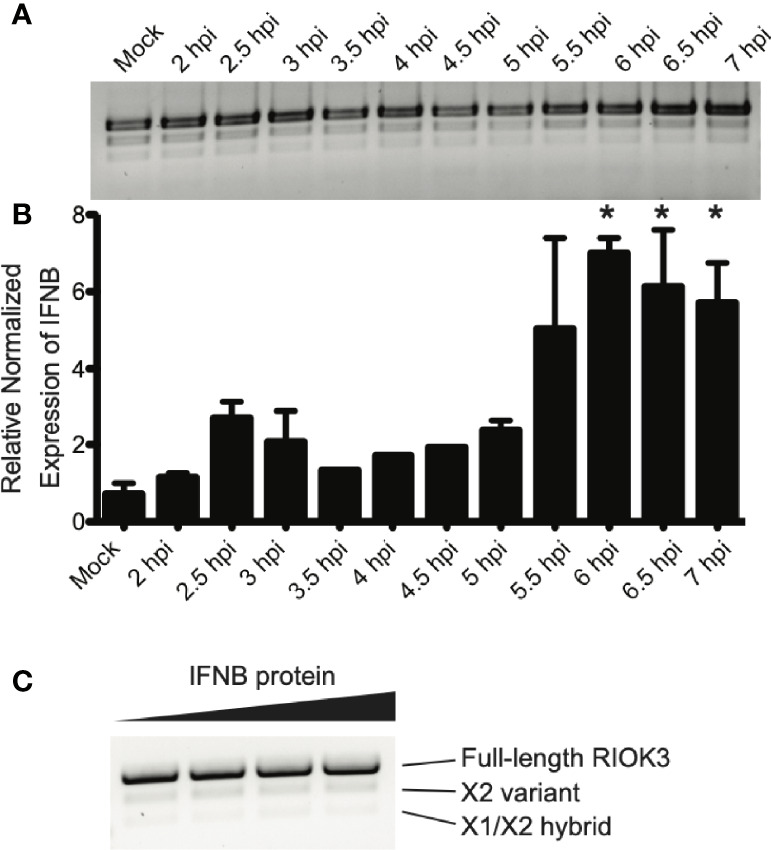
RIOK3 splicing occurs after IFNB activation, and is not affected by IFNB protein. **(A)** RIOK3 alternative splicing over the first 7 hours of RVFV MP-12 infection in HEK293 cells at MOI = 1, as measured by RT-PCR. **(B)** IFNB expression measured by qPCR at each timepoint corresponding to RIOK3 alternative splicing in response to RVFV MP-12 infection in the same HEK293 cells as in **(A)**. Asterisks indicate *p* < 0.05 compared to mock-infected cells (one-way ANOVA followed by Tukey’s HSD). **(C)** HEK293 cells in a 24-well plate were treated with 0, 0.125, 0.25, or 0.5 μg/ml of IFNB protein for 24h, and RNA was extracted. RT-PCR was performed to amplify RIOK3 mRNA.

RIOK3 protein has a role downstream of RIG-I stimulation, which culminates in homo- and heterodimerization of IRF3/IRF7 and activation of interferon-stimulated genes including the cytokine interferon beta (IFNB) (Ning et al., 2011; Tsoi et al., 2019). Therefore, we measured IFNB production as a proxy for innate immune activation. Cells were infected with RVFV and harvested as the innate immune response was being activated. We measured IFNB production *via* RT-qPCR, and simultaneously assayed RIOK3 splicing patterns by visualizing RT-PCR products by agarose gel electrophoresis. We found that RIOK3 alternative splicing isoforms were not abundant in the first 7 hours of infection, but significant IFNB expression was detected beginning at 6 hpi (**Figures 2A, B**). These data indicated that innate immune activation preceded RIOK3 alternative splicing.

To determine whether IFNB itself could trigger RIOK3 alternative splicing, we also treated cells with IFNB protein. While IFNB is readily expressed after innate immune activation *via* RIG-I activation (Kato et al., 2006), the protein is secreted and subsequently interacts with interferon receptor complexes, initiating JAK/STAT signaling (Velazquez et al., 1992; Müller et al., 1993) and activating a separate set of genes from those expressed as a result of RIG-I activation (Qureshi et al., 1996; Mesev et al., 2019). We observed no increase in alternative splicing of RIOK3 when cells were treated with IFNB protein, indicating that the splicing event is likely unique to the initial activation of innate immunity and IFNB induction *via* RIG-I and not *via* paracrine or endocrine signaling by IFNB protein (**Figure 2C**).

### 3.3 RIOK3 Transcript Isoform Abundance Is Regulated by Splicing Factor TRA2-β

Serine-arginine-rich (SR) splicing factors are a family of splicing factors that control both constitutive and alternative splicing and can promote exon skipping or inclusion. Constitutively spliced isoforms are those that are most abundant at a cell’s basal level. All SR splicing factors have an RNA-recognition domain and a serine-arginine-rich region that interacts with spliceosomal proteins. SR splicing factors have several roles in splicing, but generally work by binding RNA and recruiting spliceosomal proteins, thereby enhancing the usage of specific splice sites (Zahler et al., 1992; Zhang and Wu, 1996). TRA2-β is an SR splicing factor that usually acts as an exonic splicing enhancer by binding to clustered (A)GAA motifs in pre-mRNA (Tacke et al., 1998; Glatz et al., 2006; Best et al., 2014).

**Figure 3:**
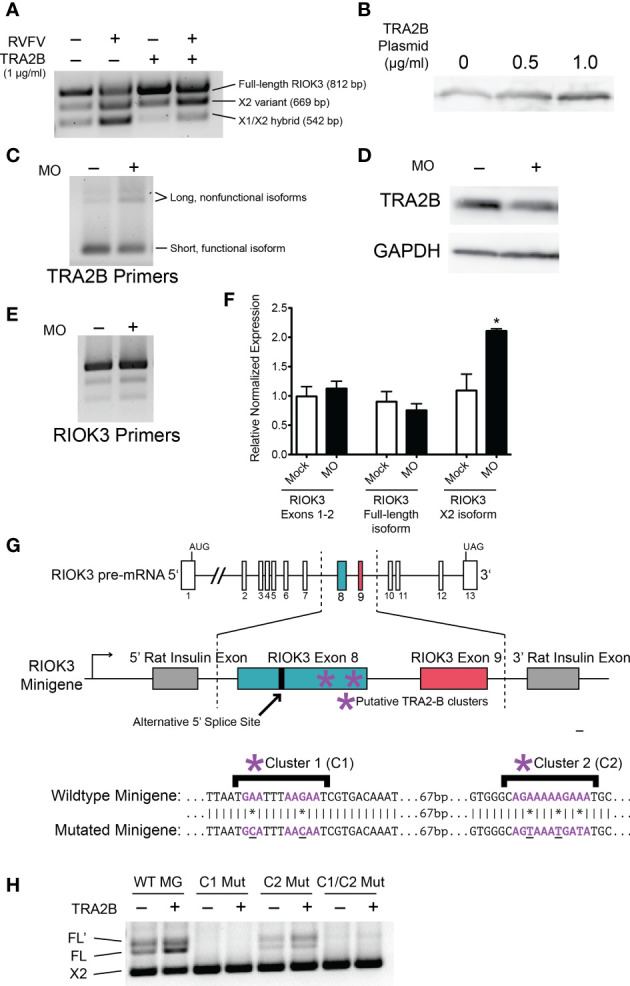
RIOK3 mRNA alternative splicing bias is controlled by TRA2-β. **(A)** HEK293 cells were transfected with TRA2-β overexpression plasmid or GFP control for 24h, then infected with RVFV MP-12 (MOI = 1) or mock for 24h before harvesting. RT-PCR shows RIOK3 splice isoforms. **(B)** HEK293 cells were transfected with TRA2-β overexpression plasmid. 24h post-transfection, cells were lysed and a western blot was performed to estimate overexpression. **(C)** HEK293 cells were treated with TRA2-β poison exon-inducing morpholino oligonucleotides (MO) at 10 μM for 24h, and RT-PCR was performed using TRA2-β primers. **(D)** HEK293 cells were treated with 5 μM MO for 24h, and lysate was used for a western blot to estimate knockdown of TRA2-β. **(E)** HEK293 cells were treated with MO as in panel C, and RT-PCR was performed using primers against RIOK3. **(F)** HEK293 cells were treated with MO as in panel C and RNA was used to perform RT-qPCR. RIOK3 exons 1-2 were used to measure overall expression of the mRNA. Each lane indicates data from biological triplicates and technical duplicates. Asterisk indicates *p* < 0.05 compared to mock transfection (Student’s t-test). **(G)** Schematic illustration of RIOK3 splicing minigene. RIOK3 exons 8 and 9 were cloned into pSpliceExpress vector, and primers spanning the splice junction between the rat insulin exon and the RIOK3 exon were used to amplify cDNA for RT-PCR. Lower panel shows mutations in putative TRA2-β binding sites. **(E)** RT-PCR using splice junction-spanning primers to amplify minigene cDNA.

We used SFmap, a web-based splicing factor motif prediction tool, to query the RIOK3 exon 8 mRNA sequence for putative splicing factors (Akerman et al., 2009; Paz et al., 2010). The SFmap algorithm uses two characteristics of splicing motifs to predict splice sites: 1) the propensity for splicing factor motifs to be in repeated clusters (Ule et al., 2006) and 2) the tendency for splice site motifs to be evolutionarily conserved (Goren et al., 2006). According to the SFmap results, RIOK3 pre-mRNA has two potential TRA2-β binding clusters. To assess whether these TRA2-β binding motifs were actively used in RIOK3 pre-mRNA splicing, we first overexpressed TRA2-β in HEK293 cells and performed RT-PCR on endogenous RIOK3 mRNA. TRA2-β overexpression paralleled an increased abundance of RIOK3 FL in mock and infected cells, indicating that exon 8 has at least one binding site that is responsive to TRA2-β protein levels (**Figure 3A**). TRA2-β protein overexpression was confirmed via western blot (**Figure 3B**).

Next, we wanted to assess whether knockdown of TRA2-β had the opposite effect as overexpression. However, siRNA is a known activator of innate immunity by activation of the RIG-I pathway (Meng and Lu, 2017; Takahashi et al., 2018). Furthermore, RIOK3 has been shown to be alternatively spliced due to RIG-I activation alone (Havranek et al., 2021). Therefore, we could not use siRNAs to decouple RIG-I-caused RIOK3 splicing from siRNA-caused RIOK3 splicing. Instead, we administered a morpholino oligo (MO; GeneTools LLC) designed to obscure an hnRNPA1 binding site on TRA2-β pre-mRNA. Binding of hnRNPA1, a splicing silencer, normally promotes exclusion of a poison exon and results in normal expression of TRA2-β (Leclair et al., 2020). Occlusion of the binding site results in inclusion of the poison exon and rapid degradation of the mRNA by nonsense-mediated decay (Stoilov et al., 2004; Leclair et al., 2020). MOs are highly stable oligonucleotide mimics that are not degraded by cellular machinery, do not elicit antiviral responses, and can be used to occlude splice sites or protein-binding motifs (Summerton, 2007; Regis et al., 2013). Upon treating cells with the TRA2-β knockdown MO, we observed a modest increase in TRA2-β poison exon expression (**Figure 3C**), a decrease in TRA2-β protein (**Figure 3D**), and a resulting increase in RIOK3 X2 (**Figure 3E**). We confirmed the latter results through RT-qPCR targeting RIOK3; while RIOK3 overall expression (measured by constitutively spliced exons 1-2) did not change, we saw a small but insignificant decrease in FL and a statistically significant 2-fold increase in X2 after treating with MO (**Figure 3F**). It is worth noting that because both the TRA2-β poison exon transcript and the RIOK3 X2 transcript are expected to be rapidly degraded by nonsense mediated decay, their actual (transient) abundance was likely higher than measured by RT-qPCR.

We next constructed RIOK3 splicing minigenes to assess the importance of the putative TRA2-β binding sites (highlighted in **Figure 3G**) that could impact RIOK3 splicing. The minigene was constructed using pSpliceExpress, a previously described backbone, which contains rat insulin exons (Kishore et al., 2008), between which we inserted RIOK3 exons 8 and 9, intron 8, and 448 bp (upstream) and 250 bp (downstream) of the surrounding intronic region. We used primers that overlapped the rat insulin exons and RIOK3 exons to isolate spliced mRNA derived solely from the minigene. We constructed a wildtype-like minigene, two mutant minigenes with nucleotides in the putative TRA2-β binding clusters C1 or C2 mutated, and a fourth minigene mutated in both C1 and C2 (**Figure 3G**). An increase in FL RIOK3 was observed when we transfected cells with the TRA2-β expression construct, and mutation of putative TRA2-β sites caused complete (C1) to partial (C2) loss of FL RIOK3 mRNA (**Figure 3H**). We also observed FL’, an isoform derived from a cryptic splice site only observed in the minigene, indicating that some of the normal splice-specific context may be missing from the minigene. Indeed, a weak splice site downstream of the FL splice site is predicted bioinformatically [NetGene2; (Brunak et al., 1991; Hebsgaard et al., 1996)]. The fact that this longer species does not appear during endogenous RIOK3 splicing suggests that a strong splicing factor binding site outside the region cloned into the minigene is responsible for avoiding FL’ in normal cells. The fact that the FL’ abundance mirrors that of FL demonstrates that it is subject to the same upstream splicing regulation as FL. These data demonstrate that splicing patterns of the RIOK3 minigenes are exquisitely sensitive to the presence of TRA2-β as well as the TRA2-β recognition sequences found in the cellular RIOK3 pre-mRNA.

**Figure 4:**
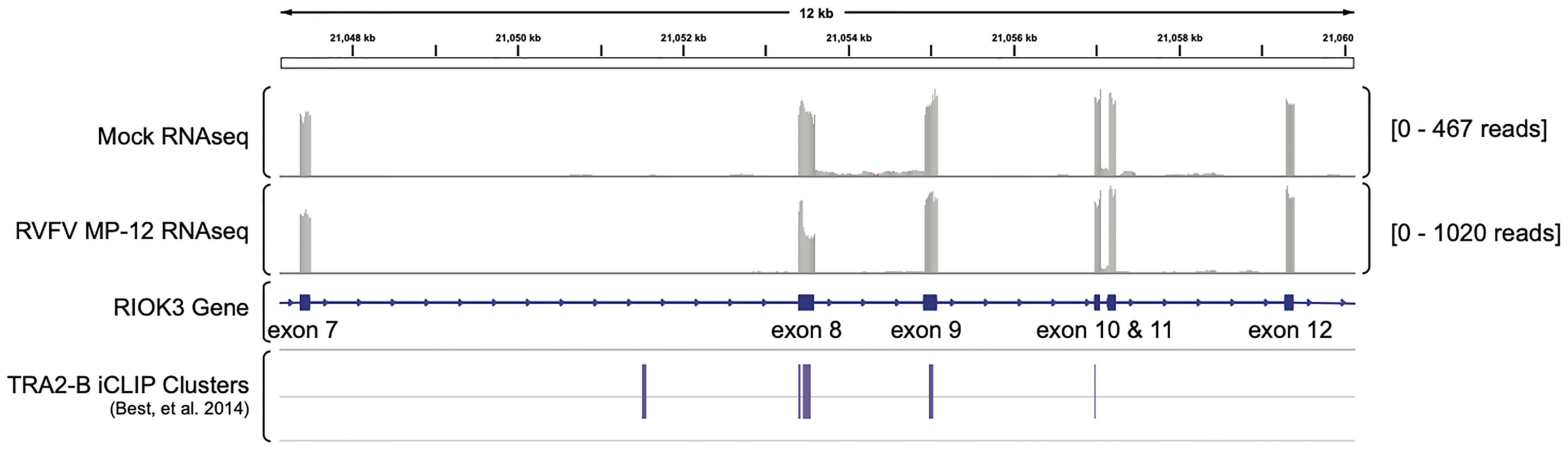
TRA2-β interacts with RIOK3 mRNA. RNAseq-derived Integrated Genomics Viewer (IGV) tracks of mRNA expression in mock- vs. RVFV MP-12-infected HEK293 cells highlighting RIOK3 (data are from Havranek et al., 2019). TRA2-β-bound iCLIP cluster reads from Best, et al. (2014; GEO accession number GSE59335) are aligned below.

Lastly, we wanted to ask whether TRA2-β protein was present at the splice site. We investigated previously published iCLIP data by Best, et al. (GEO dataset GSE59335) in which the authors sequenced and analyzed RNA bound to TRA2-β protein (Best et al., 2014). Using Integrated Genomics Viewer (Robinson et al., 2011) to visualize alignments from our previously published RNAseq data (Havranek et al., 2019), we found TRA2-β clusters in RIOK3 exon 8 in the iCLIP data, supporting our hypothesis that RIOK3 constitutive splicing is controlled by TRA2-β (**Figure 4**).

### 3.4 SR Splicing Factor TRA2-β mRNA Is Alternatively Spliced During RVFV Infection

**Figure 5:**
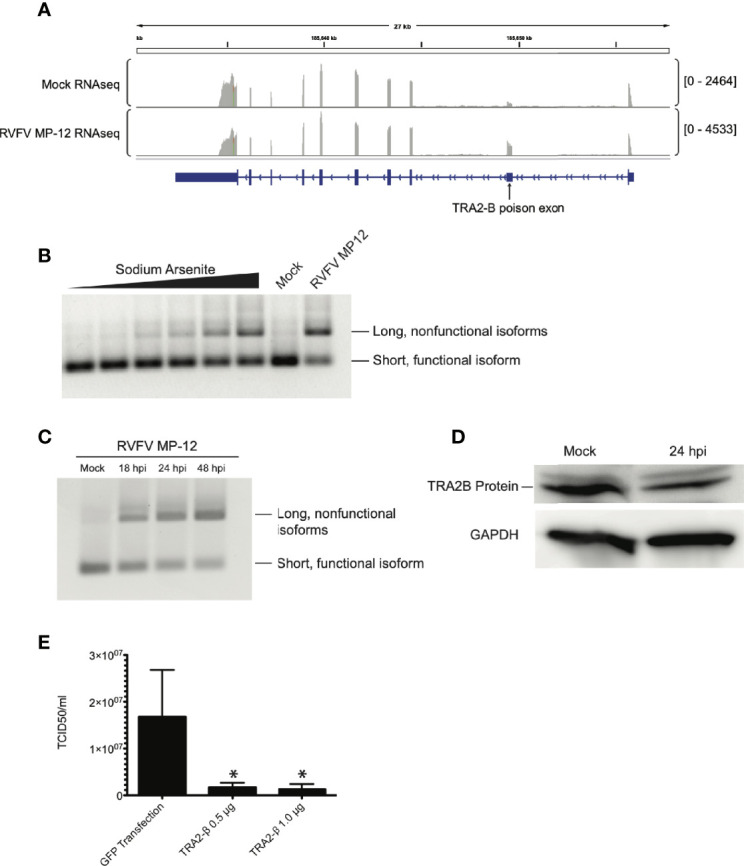
TRA2-β mRNA is alternatively spliced in response to RVFV infection. **(A)** IGV tracks of mock- vs. RVFV MP-12-infected HEK293 cells at MOI = 1, highlighting TRA2-β. TRA2-β poison exon is labeled. **(B)** HEK293 cells were treated with sodium arsenite in increasing amounts (0, 1, 10, 25, 50, or 100 μM) or infected with RVFV MP-12 for 24h. RT-PCR with primers targeting TRA2-β mRNA was visualized by agarose gel. **(C)** HEK293 cells were infected with RVFV MP-12 at MOI = 1 for the times indicated, and RT-PCR against TRA2-β was performed. **(D)** HEK293 cells were infected with RVFV for 24h at MOI = 1, then cells were lysed and visualized *via* western blot. **(E)** TCID_50_ results obtained by infecting naïve Vero cells using RVFV MP-12 particles obtained from HEK293 cells transfected with indicated plasmids and subsequently infected with RVFV MP-12 at MOI = 1. Asterisks indicate *p* < 0.05 compared to GFP transfection (one-way ANOVA followed by Tukey’s HSD).

TRA2-β protein regulates its own expression by binding to TRA2-β pre-mRNA in an auto-regulatory feedback loop (Stoilov et al., 2004). When TRA2-β protein is (over-) abundant, it binds to its pre-mRNA and forces the inclusion of an alternatively spliced non-coding “poison” exon in the mature mRNA, which leads to rapid degradation of the mRNA through nonsense-mediated decay. Dysregulation of this system can disrupt constitutive splicing in cells; some mutations that disrupt poison exons can cause disease such as cancer (Yoshida et al., 2011; Liang et al., 2018). Additionally, poison exons are present and conserved in all members of the SR splicing factor family (Lareau et al., 2007; Ni et al., 2007; Leclair et al., 2020). We previously observed that the balance of TRA2-β splicing isoforms was skewed toward the poison exon-containing isoform in an RNAseq study on RVFV MP-12-infected cells (Havranek et al., 2019) (**Figure 5A**).

We infected HEK293 cells with RVFV MP-12 and observed TRA2-β mRNA splicing *via* RT-PCR, and compared the splice pattern to that caused by sodium arsenite, a treatment that was previously shown to induce TRA2-β poison exon inclusion in response to oxidative stress (Akaike et al., 2014). In RVFV-infected cells, the TRA2-β splicing pattern was altered to favor the longer isoform which includes the poison exon (**Figure 5B**). We observed a time-dependent accumulation of the poison exon isoform upon longer infection of RVFV MP-12, up to 48 hpi (**Figure 5C**). This was supported by our observation of a decrease in endogenous TRA2-β protein in infected cells (**Figure 5D**). Next, because we previously reported that RIOK3 overexpression was detrimental to RVFV replication (Havranek et al., 2021), we asked whether TRA2-β overexpression, which would increase FL RIOK3 mRNA in cells, would also reduce RVFV MP-12 infection. In support of this hypothesis, we observed a decrease in extracellular viral particles measured by TCID_50_ assay (**Figure 5E**). Taken together, these results strongly suggest that constitutive splicing of RIOK3 by TRA2-β and/or suppression of alternative splicing of RIOK3 lead to a stronger antiviral response against RVFV infection in culture.

## 4 Discussion

We previously demonstrated that RIOK3 plays an important role in the cellular response to RVFV infection. In this study, we examined RIOK3 alternative splicing in the context of RVFV infection and observed that splice site selection in this gene is at least partially controlled by the exonic splicing enhancer TRA2-β. Interestingly, TRA2-β itself is also alternatively spliced during RVFV infection, which likely affects its expression.

RIOK3 is a still incompletely understood member of the atypical protein kinase subfamily whose functions have been variously associated with ribosome assembly, erythrocyte maturation, cellular immunity, and the hypoxia response (LaRonde-LeBlanc and Wlodawer, 2005; Shan et al., 2009; Zhang et al., 2011; Baumas et al., 2012; Feng et al., 2014; Singleton et al., 2015; Takashima et al., 2015). In different cellular and viral contexts, its expression appears to be important for either activation (Feng et al., 2014; Willemsen et al., 2017; Havranek et al., 2021) or deactivation (Takashima et al., 2015; Willemsen et al., 2017; Shen et al., 2021) of the cellular immune response. RIOK3 mRNA was found to be alternatively spliced during RVFV infection (Havranek et al., 2019; Havranek et al., 2021), and in this work we demonstrated that this alternative splicing event is observed in both human and monkey cell types, suggesting that alternative splicing at this site is evolutionarily conserved. We previously demonstrated that constitutive RIOK3 splicing is required for productive expression of IFNB, and we hypothesize that RIOK3 alternative splicing may be a mechanism to regulate the innate immune response. We also showed that RIOK3 alternative splicing is only activated by primary innate immune activation and not activated by downstream cytokine (IFNB) signaling that may occur *via* autocrine or paracrine activation of interferon receptors. These data, in conjunction with our previously reported data using RIG-I and MDA5 agonists (Havranek et al., 2021), indicate that the alternative splicing event is triggered in the early stages of the activation of the antiviral state *via* RIG-I or MDA5 and is not a feedback mechanism triggered by IFNB.

Regulation of cellular immunity at the splicing level by expression of an alternative splice isoform, either nonfunctional or inhibitory towards its normal binding partners, is an established mechanism in other innate immune proteins, possibly to prevent overstimulation of the innate immune response and cell death. For example, a short isoform of RIG-I missing its CARD domain, required for TRIM25 binding, K-63 ubiquitination and activation of RIG-I signaling, is expressed within 24h after Sendai virus infection (Gack et al., 2008). Likewise, a truncated version of TBK-1 that binds to RIG-I, but not the downstream interactor MAVS (also known as VISA, CARDIF, and IPS-1), is expressed as early as 6h after infection of Sendai virus, effectively disrupting the innate immune response (Deng et al., 2008). For MAVS, another immune signaling protein downstream of RIG-I, alternative splice isoforms coding for proteins that are unable to interact with TRAF proteins required for IFNB expression are important for modulating immune activation (Lad et al., 2008). Another example of alternative splicing regulating the immune response is in the OAS1g gene, which codes for the potent innate immune activator 2’-5’ oligoadenylate synthetase, where an alternative splice site leading to transcripts degraded by nonsense mediated decay is used to prevent excess apoptosis (Frankiw et al., 2020). An opposite mechanism exists for IRF3, where instead of expressing an inactive alternative isoform, an SR splicing factor (SRSF1) is responsible for constitutive splicing of mRNA, which results in immune activation (Guo et al., 2013). In this context, RIOK3 joins a rich family of proteins associated with the antiviral state that are regulated *via* splicing.

Here, we demonstrated that splicing of RIOK3 pre-mRNA is at least partially controlled by TRA2-β, which implicates this splicing factor as a regulator of one facet of the innate immune response. TRA2-β is one of 12 members of the SR splicing factor family that form a tightly co-regulated web to correctly splice mRNA (Long and Caceres, 2008; Leclair et al., 2020). Each SR splicing factor has at least one poison exon, which is in turn regulated by other SR spicing factors. It is likely that SR splicing factors are required for the alternative splicing that occurs during viral infection, but the exact roles of each SR protein during infection remain unclear. To our knowledge, TRA2-β has not been implicated in regulation of the immune response, but has been reported to be a constitutive splicing factor involved in the splicing of a diversity of mRNAs during normal cellular function and development (Hofmann et al., 2000; Grellscheid et al., 2011; Elliott et al., 2012). As an upstream factor of innate immune activation, cellular alteration of TRA2-β concentration could be an early switch for alternative splicing in response to infection.

We cannot rule out that other splicing factors might also contribute to constitutive or alternative RIOK3 pre-mRNA splicing. However, since the principal mRNA isoforms detected in all tissue types are of the FL, X1, and X2 types surrounding exons 7, 8 and 9, and regulation of this splicing is strongly impacted by levels and binding of TRA2-β, it appears that this splicing factor is the primary regulator of RIOK3 splicing. Additionally, according to our RNA seq data (Havranek et al., 2019), only two other SR splicing factors are alternatively spliced in response to RVFV MP-12 infection: TRA2-α, and SRSF11. TRA2-α is a partial paralogue to TRA2-β (Dauwalder et al., 1996), and also has an increased poison exon inclusion, and therefore likely less protein. SRSF11 encodes the protein p54 that binds to intronic C-rich regions (Kennedy et al., 1998). RIOK3 pre-mRNA does not contain intronic C-rich stretches. It is intriguing that the pre-mRNA for TRA2-β is also alternatively spliced to include a poison exon as a result of RVFV infection. As inclusion of the poison exon decreases expression of the TRA2-β protein, it is plausible that the decrease in TRA2-β concentration results in alternative splicing of RIOK3 and other mRNAs. The signal(s) that trigger alternative splicing of TRA2-β pre-mRNA during viral infection will be an intriguing avenue of investigation. One intriguing possibility is through the TRAF6-mediated K-63 ubiquitination and nuclear localization of hnRNPA1 during innate immune activation (Culver-Cochran and Starczynowski, 2018); if the activity of TRAF6 is inhibited during infection hnRNPA1 activity could drop, leaving TRA2-β pre-mRNA vulnerable to inclusion of the poison exon. TRAF6 is known to be targeted for degradation during flavivirus infection (Chan et al., 2016; Dainichi et al., 2019). Additionally, it is possible that a general reduction in transcription caused by viral protein NSs (Billecocq et al., 2004) could be responsible for decreased expression of RIOK3 and TRA2-β mRNA. However, we noted an increase in mRNA read abundance of these transcripts in our RNAseq study (Havranek et al., 2019), indicating either that RIOK3 and TRA2-β mRNA abundance is not affected by NSs transcriptional shutdown in the time frame of acute infection, and/or that the relative abundances of splicing isoforms of these mRNAs exerts a more powerful effect than their absolute copy numbers.

It will be of interest to examine how RIOK3 alternative splicing causes a reduction in virus-induced IFNB production, either through the expression of a truncated protein encoded by the X2 isoform, or through reduction of the intracellular levels of constitutively spliced mRNA, or both. Additionally, the apparently conflicting results of RIOK3’s reported activity in different cell types and during infection with different viruses suggests that RIOK3’s role depends on the antiviral pathways that are activated and inhibited during infection, which may also depend on the isoforms of RIOK3 that are expressed in response to infections with different viruses. Finally, TRA2-β’s ability to regulate alternative splicing and the fact that it is prone to alternative splicing itself emphasize its potential to act as a central regulator during the cellular antiviral immune response. We anticipate that documentation of TRA2β’s roles during these dynamic events in the cell will continue to expand.

## Data Availability Statement

Publicly available datasets were analyzed in this study. This data can be found here: https://www.ncbi.nlm.nih.gov/pmc/articles/PMC6538246/; https://www.ncbi.nlm.nih.gov/geo/query/acc.cgi?acc=GSE59335. All other data used in this study are available on request.

## Author Contributions

LW: Conceptualization, Investigation, Data Curation, Formal Analysis, Visualization, Writing – Original Draft Preparation. TB: Investigation, Data Curation. HG: Investigation, Data Curation. MH: Conceptualization, Investigation, Data Curation. J-ML: Conceptualization, Investigation, Visualization. JSL: Conceptualization, Supervision, Writing – Review & Editing.

## Funding

We gratefully acknowledge funding for this research from NIH grants R03AI137620, R03TR002937 to JSL, P20GM103546 (B. Bowler, P.I.; JSL Pilot Project P.I.), NIH ASCEND Grant 4UT2GM130166-02 (Subaward GM130166 to Lodmell, J.S.), and matching funds through the UM Center for Translational Medicine, and NIH R41TR003929 (F. Astruc-Diaz, P.I.).

## Conflict of Interest

The authors declare that the research was conducted in the absence of any commercial or financial relationships that could be construed as a potential conflict of interest.

## Publisher’s Note

All claims expressed in this article are solely those of the authors and do not necessarily represent those of their affiliated organizations, or those of the publisher, the editors and the reviewers. Any product that may be evaluated in this article, or claim that may be made by its manufacturer, is not guaranteed or endorsed by the publisher.

## Supplementary Material

Supplementary Figure 1 HEK293 cells were infected with RVFV at MOI = 1 for the times indicated, then cells were lysed and visualized by western blot (see *Materials and Methods*).
