# Supplementary figures and images for: Tra2beta-Dependent Regulation of RIO Kinase 3 Splicing During Rift Valley Fever Virus Infection Underscores the Links Between Alternative Splicing and Innate Antiviral Immunity
Source: Front Cell Infect Microbiol. 2022 Jan 19;11:799024. doi: 10.3389/fcimb.2021.799024 (PMC8807687; doi:10.3389/fcimb.2021.799024)

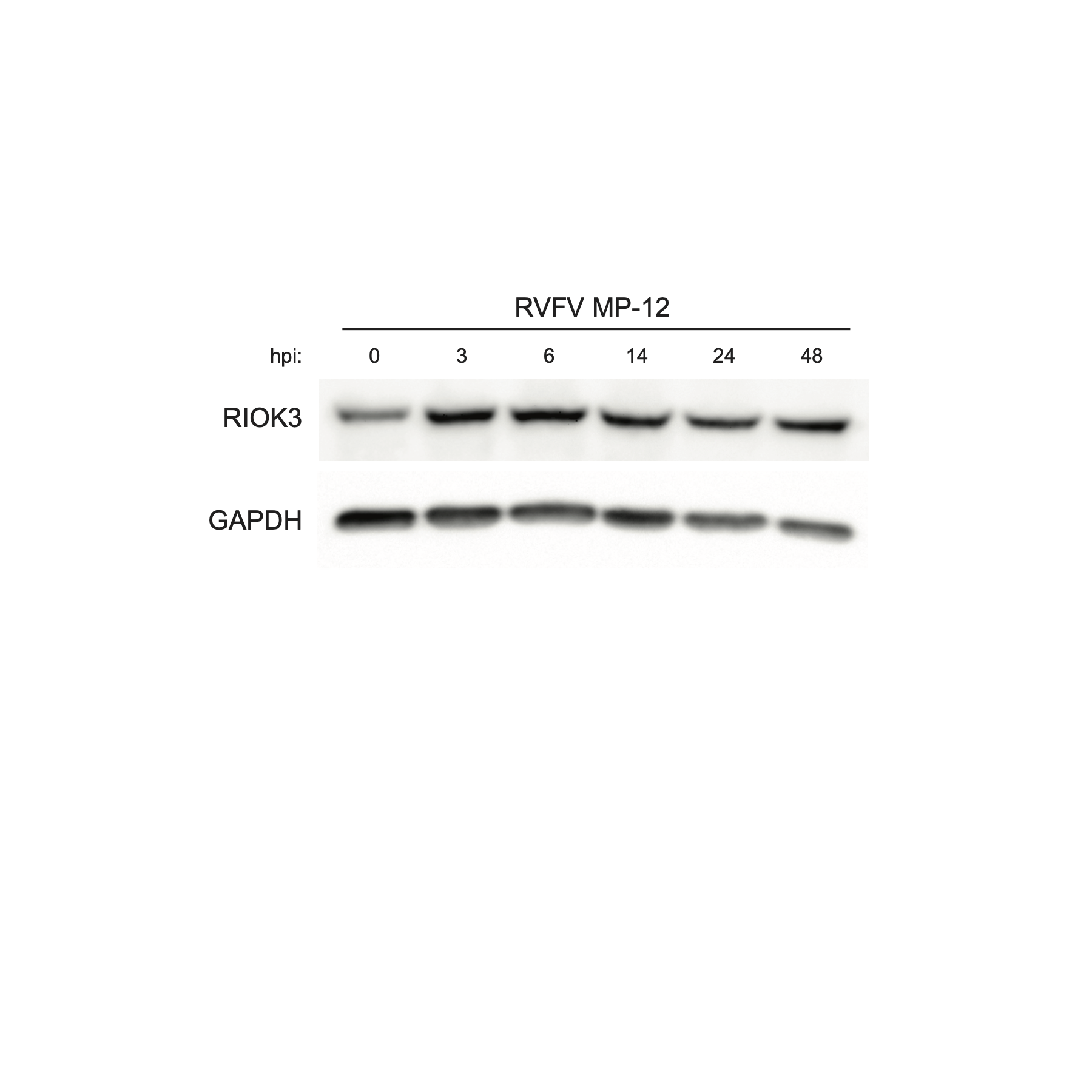

Supplement: Supplementary Figure 1 — HEK293 cells were infected with RVFV at MOI = 1 for the times indicated, then cells were lysed and visualized by western blot (see Materials and Methods). [file Image_1.tiff]
